# Physiological, Anatomical and Metabolic Implications of Salt Tolerance in the Halophyte *Salvadora persica* under Hydroponic Culture Condition

**DOI:** 10.3389/fpls.2016.00351

**Published:** 2016-03-22

**Authors:** Asish K. Parida, Sairam K. Veerabathini, Asha Kumari, Pradeep K. Agarwal

**Affiliations:** ^1^Division of Plant Omics, Council of Scientific and Industrial Research-Central Salt and Marine Chemicals Research InstituteBhavnagar, India; ^2^Academy of Scientific and Innovative Research, Council of Scientific and Industrial Research-Central Salt and Marine Chemicals Research InstituteBhavnagar, India

**Keywords:** halophyte, organic metabolites, palisade, *Salvadora persica*, spongy parenchyma, sodium, stomata, xylem

## Abstract

Salt tolerance mechanism of an extreme halophyte *Salvadora persica* was assessed by analyzing growth, nutrient uptake, anatomical modifications and alterations in levels of some organic metabolites in seedlings imposed to various levels of salinity (0, 250, 500, and 750 mM NaCl) under hydroponic culture condition. After 21 days of salt treatment, plant height, leaf area, and shoot biomass decreased with increase in salinity whereas the leaf succulence increased significantly with increasing salinity in *S. persica*. The RWC% of leaf increased progressively in salt-treated seedlings as compared to control. Na^+^ contents of leaf, stem and root increased in dose-dependent manner whereas there was no significant changes in K^+^ content. There was significant alterations in leaf, stem, and root anatomy by salinity. The thickness of epidermis and spongy parenchyma of leaf increased in salt treated seedlings as compared to control, whereas palisade parenchyma decreased dramatically in extreme salinity (750 mM NaCl). There was a significant reduction in stomatal density and stomatal pore area of leaf with increasing salinity. Anatomical observations of stem showed that the epidermal cells diameter and thickness of cortex decreased by salinity whereas thickness of hypodermal layer, diameter of hypodermal cell, pith area and pith cell diameter increased by high salinity. The root anatomy showed an increase in epidermal thickness by salinity whereas diameters of epidermal cells and xylem vessels decreased. Total soluble sugar content remained unchanged at all levels of salinity whereas reducing sugar content increased by twofold at high salinity (750 mM NaCl). The starch content of leaf decreased progressively in NaCl treated seedlings as compared to control. Total free amino acid content did not change at low salinity (250 mM), whereas it increased significantly at higher salinity (500 and 750 mM NaCl). The proline content increased in NaCl treated seedlings as compared to control. There was no significant changes in polyphenols level of leaf at all levels of salinity. The results from the present study reveal that seedlings imposed with various levels of salinity experience physiological, biochemical and anatomical modifications in order to circumvent under extreme saline environment. The vital mechanisms of salt tolerance in *S. persica* are higher accumulation of organic metabolites, increase in leaf succulency, efficient Na^+^ sequestration in the vacuole, K^+^ retention in the photosynthetic tissue and increase in WUE by reducing stomatal density. Therefore, *S. persica* is a potential halophytic species to be cultivated in saline lands to eliminate excess salt and make it favorable for agriculture.

## Introduction

Salinization of soil is one of the most serious threat to irrigated crop production in arid and semi-arid regions (Zakery-Asl et al., 2014). Salinity is a soil condition characterized by a high concentration of soluble salts. Soils are categorized as saline when the EC is 4 dS m^-1^ or more which is equivalent to approximately 40 mM NaCl and generates approximately an osmotic pressure of 0.2 MPa (Munns and Tester, 2008). Salinity induces ionic and osmotic imbalance at cellular level which results in ion toxicity and osmotic stress (Khan et al., 2000). The state of osmotic imbalance exerted on plants when they are growing on a salt marsh or other excessive saline condition is termed as salt stress. In case of glycophytes, sodium is a non- essential element affecting growth and various metabolic processes of plants that eventually cause morphological, physiological and biochemical alterations at higher doses of salinity (Parida and Jha, 2013). Salinity mainly affects the plants by reducing the water potential of the soil leading to deficiency of water availability to plants (Hasegawa et al., 2000; Hasegawa, 2013). This decrease in water availability ultimately reduces the photosynthetic rate and hence the overall growth of the plant. In order to survive in soils with high salinity, plants develop various physiological and biochemical mechanisms that include ion compartmentalization (Shabala and Mackay, 2011); biosynthesis of osmo-protectants or compatible solutes *viz*. amino acids, sugars, quaternary ammonium compounds, tertiary sulfonium compounds, polyols etc. (Slama et al., 2015); increased activation of antioxidative enzymes *viz.*, superoxide dismutase (SOD), catalase (CAT), glutathione peroxidase (GPX), glutathione *S*-transferases (GST), ascorbate peroxidase (APX), dehydroascorbate reductase (DHAR), glutathione reductase (GR), and monodehydroascorbate reductase (MDHAR) (Parida and Jha, 2010); synthesis of antioxidant compounds viz., ascorbate, glutathione (GSH), flavonoids, carotenoids and tocopherols (Shabala et al., 2012) and modulations of plant hormones (Gupta and Huang, 2014). Apart from physiological and biochemical alterations, anatomical modifications like thickening of leaf, increased epidermal thickness, changes in stomatal distribution, changes in xylem components of stem and root etc. are induced by salinity (Vijayan et al., 2008). These modifications lead to osmotic and ionic adjustment of the cells under high salinity condition. The osmotic adjustment of the cell can also be achieved by substantial and sustained intracellular compartmentalization of the salt between cytoplasm and vacuole. The increase in leaf succulence and presence of salt glands are some essential components for regulation of leaf salt concentrations in halophytic species.

Plants growing well in saline conditions are by definition called halophytes. Halophytic species, constituting 1% of the world flora (Flowers and Colmer, 2008) has the ability to grow and complete their life cycle on a substratum that contains high concentration of soluble salts (Parida and Das, 2005). Halophytes can grow in a wide variety of habitat including coastal regions and salt marshes (Shabala, 2013). The ability of halophytic species to remove salt from saline land makes it a promising desalinization tool (Shabala, 2013). Halophytic species are benefited from salinity level which is detrimental for many crop species (Shabala et al., 2012). The adaptations to the environmental stress can either be done by the pre-existing or by induced defenses (Pastori and Foyer, 2002). The mechanism of salt resistance in halophytes is usually grouped into two main categories, i.e., salt avoidance and salt tolerance (Sabovljevic and Sabovljevic, 2007). The strategy behind salt tolerance may involve certain physiological and/or biochemical alterations in the plants that enable the plant to maintain viability of protoplasm during accumulation of salt ions inside the cells. Salt tolerance in halophytes can be accomplished either by salt exclusion or salt inclusion and salt tolerant plants utilize the energy for the exclusion of excess salt in order to protect themselves from toxic effects of excess salt (Ashraf et al., 2006). Salt avoidance may involve some structural and physiological adaptations so as to exclude salt through root membrane or minimize the salt concentrations in the cell. This may involve passive exclusion of ions through permeable membrane or the active expelling of ions through ion channels. Besides physiological alternation, halophytes also adapt some anatomical modifications to cope up with high external soil salinity (Ola et al., 2012). These modifications include increase in the epidermal thickness and spongy tissue of the leaves and reduction in conducting tissue component of stem and root like xylem vessel (Hussein et al., 2012). As proposed by earlier reports, halophytes possess more salt tolerant genes than glycophytes (Askari et al., 2006; Yu et al., 2011) which are mostly involved in membrane transport, redox reactions, signal transduction, and other processes (Zhang et al., 2008).

*Salvadora persica* L. (Miswak) which belongs to the family Salvadoraceae is a desert facultative halophytic plant. It is a medium sized tree containing several bioactive compounds such as alkaloids, tannins, saponins, and sterols (Sharma and Ramawat, 2013). Owing to its medicinal properties, *S. persica* has been the object of discovery over more than three decades. It is used for oral hygiene in many parts of the world because of its antibacterial properties and ability to remove plaque (Esfahani et al., 2014). The cytotoxic effects of *S. persica* plant on human gingival fibroblast cells has been reported by Balto et al. (2014). Khan et al. (2014) have reported the hypoglycemic and hypolipidemic activities of its root extracts on diabetic rats. *S. persica* is a facultative halophyte which is adapted to survive under various adverse environmental conditions ranging from deserts to heavy soil, non-saline to highly saline soil, and dry regions to marshy and waterlogged areas (Reddy et al., 2008). In contrasts to several species of halophytes requiring fresh water for their germination, *S. persica* germinates in saline water of approximately 15 dS m^-1^ salt concentration (Rao et al., 2004). Therefore, understanding the salt tolerance mechanism of *S. persica* is vital. Some preliminary studies have been carried out on growth, mineral content and antioxidant activity in callus cultures of *S. persica* treated with very low level of salinity up to 200 mM NaCl (Sharma and Ramawat, 2013). Salinity effects on leaf gas exchange and ion accumulation have been reported in *S. persica* plant treated with moderate salinity up to 200 mM NaCl (Maggio et al., 2000). However, it can tolerate very extreme salinity of much higher than 200 mM. In our previous study, we have reported salinity induced changes in antioxidative enzymes and their role in protecting the photosynthetic machinery from oxidative damage conferring salt tolerance up to 1000 mM NaCl in *S. persica* grown in soil (Rangani et al., 2016). In the present investigation, we have first time reported an integrated study on physiological, biochemical and anatomical modifications of *S. persica* under long-term exposure to extreme salinity up to 750 mM NaCl under hydroponic culture condition to obtain an insight into the salt adaptive mechanism in this halophyte with a future aim to develop salt tolerant crops.

## Materials and Methods

### Plant Material, Growth Condition and Treatment

The seeds of *S. persica* Linn. were collected from CSMCRI salt farm area, Bhavnagar, Gujarat, India (latitude 21° 47.306′ N and longitude 72° 7.417′ E). Seedlings were raised in the experimental greenhouse under non-saline condition and were exposed to daylight with photosynthetic active radiance (PAR) of 1,000–1,250 (μmol m^-2^ s^-1^). Two months old healthy seedlings of uniform size were selected and further grown in Hoagland’s nutrient medium (pH 5.8) under hydroponic culture condition. These plants were adapted to grow hydroponically for 14 days prior to salt treatment. After adaptation period, the plants were subjected to salt treatment by supplementing the nutrient medium with varied NaCl concentrations (250, 500, and 750 mM). The control plants were grown in the nutrient medium devoid of NaCl. In a culture room, the hydroponic cultures were maintained under a 14 h d^-1^ photoperiod at 500 μmol m^-2^ s^-1^ photon flux densities with 25 ± 2°C room temperature and 60% relative humidity. The nutrient solutions were replaced with freshly prepared solutions at every 7 days intervals. After 21 days of salt treatment, leaf, stem, and root samples were harvested from control and NaCl-treated plants for estimation of various parameters. Leaves occupying the same position were sampled from control and NaCl-treated plants for estimation of various biochemical parameters.

### Measurement of Growth Parameters

Plant height, total leaf area per plant, fresh and dry biomass of leaf, stem and root of five plants from each treatment were recorded after 21 days of treatment. For measurement of fresh and dry weights of leaf, stem and roots, respective plant parts were excised from control and NaCl-treated plants and the fresh weight was noted immediately. Later, these plant parts were wrapped in pre-weighed aluminum foils and kept in an incubator at 80°C for 48 h before the dry weight was recorded. Total green leaf area per plant was measured in both control and NaCl-treated plants, using Digimizer software (version 4.3.1, MedCalc Software, Belgium).

### Estimation of Leaf Relative Water Content (RWC %)

The relative water content (RWC) of leaves of control and NaCl-treated plants was measured following the method of Barrs and Weatherley (1962). Leaf fresh weight (LFW) was immediately noted after sampling and subsequently immersed into distilled water for 8 h at room temperature. Leaves were then blotted dry and leaf turgid weight (LTW) was taken prior to incubating at 80°C for 48 h. After incubation period, leaf dry weight (LDW) was also noted. The leaf RWC was calculated using following formula:

$$RWC\,\;\%=\left[\frac{LFW-LDW}{LTW-LDW}\right]\times 100.$$

### Estimation of Mineral Ion Contents

Mineral ion contents were determined using dried leaf, stem, and root samples. The excised samples were dried in an oven at 80°C for 48 h. After drying, pre-weighed samples (about 0.5 g) were homogenized and placed in a 25 mL volumetric flask. The flasks containing samples were placed on the hot plate after adding 10 mL acidic mixture of HNO_3_ and HClO_4_ (9:4) in a fume hood chamber and digested until the liquid became colorless and production of red NO_2_ fumes ceased. Further, the contents were evaporated to reduce the volume to 3–5 mL. After cooling the volumetric flasks to room temperature, volume was made up to 25 mL by adding 20 mL deionised water. The solutions were filtered through Whatman No. 1 filter paper and stored. Aliquots of this solution were used for the determination of ions *viz.*, Na, K, Ca, Mg, Mn, Zn, Fe, and B content of leaf, stem and root by Inductively Coupled Plasma Atomic Absorption Spectrometry (Optima 2000DV, Perkin Elmer, USA).

### Measurement of Anatomical Parameters of Leaf, Stem, and Root

Salinity induced anatomical adjustments were deduced by sectioning leaf, stem, and root tissues under the light microscope (Leica Microsystems Ltd., version 3.0.0, Switzerland) using a cleaned razor blade (Gillette). The sections from control and NaCl-treated plants were stained separately with safranin and fast-green following the method described by the Schuerger et al. (1997). The finest leaf sections were selected and stained initially with the safranin for 2 min, then stained with fast-green for another 2 min followed by fixing on the glass slide containing a drop of glycerol and finally covered with a cover glass slip. Similarly, stem and root sections were performed but stained only with fast-green for 2 min followed by the fixation on slide. All sections were observed under Bright-field and Dark-field Compound microscope (Olympus BX51, Tokyo, Japan) at 10X, 20X, 40X, and 100X magnifications. The measurements of thickness of upper epidermis, palisade layer, spongy parenchyma layer, lower epidermis, pith diameter, thickness of cortex, hypodermis, epidermal cells diameter, hypodermal cells diameter, cortex cells diameter, xylem vessels diameter and pith cells diameter were measured using Image Pro Analyser software (Olympus, Tokyo, Japan).

To study the salinity induced changes in stomatal characteristics, leaf tissues from control and NaCl-treated plants were procured and leaf impression was made by applying nail polish on the upper and lower surfaces of the leaf and allowed it to dry. Later, a one sided cello tape was stick on the applied surface and pealed slowly so that the applied nail polish was completely peeled off the leaf surface and fixed on the glass slide for observation. The leaf impression was observed under Bright and Dark fields of compound microscope (Olympus BX51, Tokyo, Japan) at 10X, 20X, 40X, and 100X magnifications. The measurements of stomatal pore diameter, pore area, number of stomata per unit area and stomatal density were undertaken using Image Pro Analyser software (Olympus, Tokyo, Japan). Stomatal index (SI) was calculated using the formula given below:

$$SI\left(\%\right)=\left[\frac{\left(Stomatal\,\;density\right)}{\left(Stomatal\,\;density+Epidermal\,\;cell\,\;density\right)}\right]\times 100$$

### Extraction of Samples for Estimation of Various Metabolites

The leaf tissue from control and NaCl-treated plants were dried in an incubator at 80°C for 72 h. The dried tissue were weighed and subsequently ground to powder in ball mill (Fritsch Pulverisette 7 Micro Mill, Germany). The powder was dissolved in 10 mL of 80% warmed ethanol and vortexed. These ethanolic extracts were then centrifuged at 10,000 × *g* for 10 min at room temperature and supernatant was collected. The pellet was re-extracted twice with 80% warmed ethanol and the supernatants were pooled. About10 mL of ethanolic extract prepared above was evaporated to dryness and the residues were re-dissolved in 10 mL of MilliQ water and subsequently kept on rotary shaker for 2 h. The solutions were then filtered using Whatman no. 1 filter paper and the filtrate was used for the estimation of total soluble sugar, reducing sugars and total polyphenol analysis and the pellet was processed further for estimation of starch. The pellet left after the ethanolic extraction, was suspended in 10 mL pre-chilled 52% (v/v) perchloric acid. The acidic suspension was then centrifuged at 10,000 × *g* for 10 min at 4°C and the supernatant was collected. Re-extracted the pellet twice with 52% perchloric acid and the supernatants were pooled. The supernatant collected was used for the estimation of starch.

### Estimation of Total Soluble Sugars

Total soluble sugar content was estimated following the method described by Hansen and Møller (1975). About 10 mL of ethanolic extract prepared earlier was evaporated to dryness and the residues were re-dissolved in 10 mL of milliQ water and subsequently kept on rotary shaker for 2 h. The solutions were then filtered using Whatman no. 1 filter paper and the filtrates were diluted to 10-fold using MilliQ water, for the estimation of total soluble sugar. Five hundred microliter of diluted sample was taken to which 2 mL of 0.2% (w/v) pre-chilled anthrone-sulphuric acid reagent was added and vortexed. The samples were boiled for 10 min and allowed to cool on ice bath and the absorbance was taken at 630 nm using a microplate reader (Epoch^TM^, BioTek, USA). The total sugar content was estimated from a standard curve plotted using 0–100 μg of glucose.

### Estimation of Reducing Sugars

Reducing sugars content was estimated by alkaline copper method using arsenomolybdate reagent as described earlier by Green et al. (1989). The alkaline copper tartarate (ACT) reagent was prepared by mixing alkaline tartarate reagent (2.5% Na_2_CO_3_, 2% NaHCO_3_ and 2.5% sodium potassium tartarate) with copper reagent (15% CuSO_4_ containing a drop of H_2_SO_4_) in the ratio of 24:1. To prepare arsenomolybdate reagent, 2.5 g ammonium molybdate was dissolved in 45 mL water with addition of 2.5 mL H_2_SO_4_. Finally, added 0.3 g disodium hydrogen arsenate dissolved in 25 mL water to the mixture and stored. Meanwhile, 10 mL of ethanolic extract prepared earlier was evaporated to dryness followed by cooling at room temperature. The aliquots (2 mL) of water extracts prepared above, was taken in a test tube, to which 1 mL ACT was added and vortexed. The reaction mixture was then boiled for 10 min and cooled. Lastly, 1 mL of arsenomolybdate reagent was added and vortexed and absorbance was recorded at 510 nm. The reducing sugar content was determined from a standard curve prepared against pure glucose (0–50 μg).

### Estimation of Starch

Starch content was extracted and estimated by following the method described by Hansen and Møller (1975). The perchloric acid extracts (PAE) prepared earlier were diluted to 20-fold with MilliQ water, prior to starch estimation. Five hundred microliter of diluted PAE were dispensed in test tubes kept on ice bath. To which, 2 mL of 0.2% pre-chilled anthrone-sulphuric acid reagent was added and vortexed. The reaction was initiated by keeping the samples on a boiling water bath for 10 min. The boiled samples were then kept on ice bath to terminate the reaction. The absorbance was recorded at 630 nm using microplate reader (Epoch^TM^, BioTek, USA). To determine the starch concentration, a standard curve was plotted with 0–100 mg of glucose and the obtained value was multiplied by 0.9 for conversion of glucose value to starch.

### Estimation of Total Free Amino Acids

Total free amino acids were extracted and determined following the methods described earlier by Yemm et al. (1955). Ten mililiter of ethanolic extract prepared earlier was evaporated to dryness and the residues was dissolved in 10 mL of 0.2 M sodium citrate buffer (pH 5.0) and subsequently kept on rotary shaker for 2 h. The solutions were then filtered using Whatman no. 1 filter paper and the filtrate was used for estimation of total amino acid. One mililiter of above filtrate was taken to which 1 mL of 4% ninhydrin reagent prepared in methyl cellosolve and 0.2 M sodium acetate buffer in the ratio of 1:1 was added to it and vortexed. The samples were finally boiled for 20 min and then cooled. The volume was made up to 10 mL with MilliQ water. Absorbance was taken at 570 nm and a standard curve was plotted with glycine (0–100 μg) to calculate total free amino acids.

### Estimation of Proline

The leaf sample (0.5 g) from control and NaCl-treated plants were extracted in 3% sulfosalicylic acid and the proline content was estimated following the method of Ringel et al. (2003) using acid-ninhydrin reagent. The pre-weighed leaf samples were homogenized in 5 mL of freshly prepared 3% (w/v) sulfosalicylic acid followed by centrifugation at 15000 × *g* for 10 min. Supernatant (1 mL) was collected in a test tube and to this added 1 mL of acid-ninhydrin reagent and 1 mL of glacial acetic acid. To initiate the reaction, the mixture was boiled at 99°C for 1 h. After 1 h, the reaction was terminated by immediately keeping the test tubes in an ice bath. After a while, 2 mL of toluene was added to it and vortexed for about 10–20 s. The reacted mixture was then allowed to settle down until the mixture appears bi-phasic. The chromophore containing toluene was aspirated from aqueous phase into fresh vial. Two hundred microliter aliquots of toluene extracts were transferred into a 96 well microplate and absorbance was taken at 520 nm using microplate reader (Epoch^TM^, BioTek, USA). The proline content was calculated from a standard curve prepared against L-Proline (0–100 μg).

### Determination of Total Polyphenol

Total polyphenols were determined according to the procedures of Chandler and Dodds (1983). Briefly, 10 mL of ethanolic extract prepared earlier was evaporated to dryness. The residue was dissolved in 10 mL of MilliQ water and subsequently kept on rotary shaker for 2 h. The solution was then filtered using Whatman no. 1 filter paper. One mililiter of the filtrate was taken in a test tube, to which added 2 mL MilliQ water and 1 N Folin–Ciocalteu’s reagent and incubated for 3 min at room temperature after vortexing. Later, 2 mL of freshly prepared 20% Na_2_CO_3_ was added to each tube and mixed thoroughly. The solution was boiled for 1 min in a water bath and cooled. Finally, MilliQ water was added to make up the volume of the solution to 10 mL and absorbance was recorded at 650 nm using a microplate reader (Epoch^TM^, BioTek, USA). A standard curve was prepared using 10–100 μg of catechol (Sigma). From the standard curve, the concentrations of phenols in the unknown samples were calculated.

### Statistical Analysis

All the experiments were conducted with a minimum of three replicates and the results were expressed as mean ± standard deviation (SD). All the data were subjected to one-way analysis of variance (ANOVA) and Duncan’s multiple-range test (*P* ≤ 0.05) using the Sigma Plot v12.0 statistical software (Systat Software Inc., Chicago, IL, USA).

## Results

### Effects of Salinity on Growth and Morphology

**FIGURE 1:**
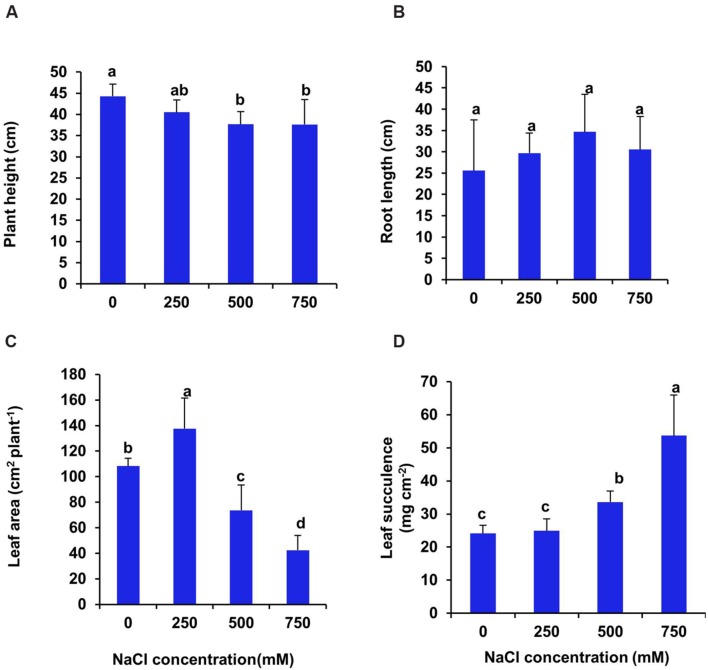
**Salinity induced changes in growth parameters of *S. persica* after 21 days of treatment under hydroponic condition. (A)** Plant height; **(B)** Root length; **(C)** Leaf area and **(D)** Leaf succulence. The values are mean ± SD (*n* = 5). The different letters on the top of the error bars indicate statistically different means at *P* ≤ 0.05.

The seedlings of *S. persica* withstand salinity level up to 750 mM NaCl for 21 days under hydroponic culture condition without any lethal effects. The salinity induced modulations of growth were assessed by analyzing plant height, leaf area and fresh and dry biomass of leaf, stem and root. After 21 days treatment, there was no significant changes in plant height between control and 250 mM NaCl treated plants. In contrasts, the plant height declined significantly by 15.6% in plants treated with high salinity of 500–750 mM NaCl (**Figure 1A**). The root length of *S. persica* remained unchanged at all levels of salinity (**Figure 1B**). The total leaf area increased significantly by 27% at low salinity (250 mM NaCl) and then decreased by 31.9 and 60.7% respectively in 500 and 750 mM NaCl treated plants as compared to control (**Figure 1C**). The succulency of leaf remained unchanged between control and 250 mM NaCl treated plants. However, leaf succulence increased significantly by 40 and 124% respectively in 500 and 750 mM NaCl treated plants as compared to control (**Figure 1D**).

**FIGURE 2:**
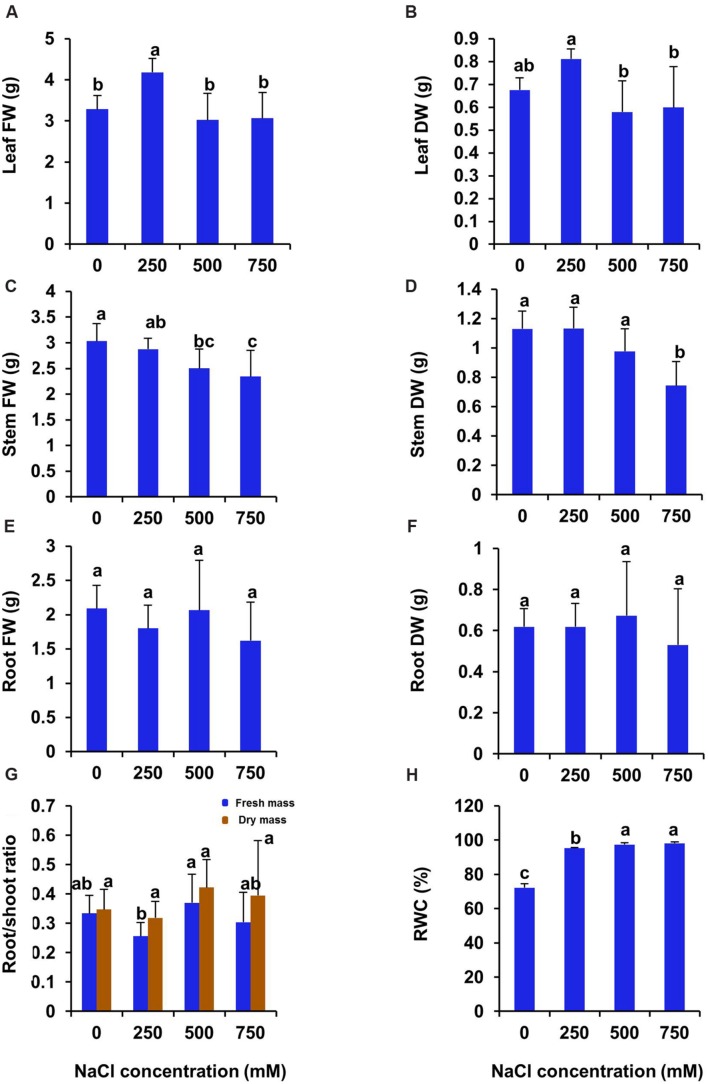
**Salinity induced changes in biomass and leaf relative water contents in *S. persica* after 21 days of salt treatment. (A)** Leaf FW; **(B)** Leaf DW; **(C)** Stem FW; **(D)** Stem DW; **(E)** Root FW; **(F)** Root DW; **(G)** Root/shoot ratio and **(H)** RWC (%). The values are mean ± SD (*n* = 5). The different letters on the top of the error bars indicate statistically different means at *P* ≤ 0.05.

After 21 days of treatment, both fresh and dry biomass of leaf increased significantly in 250 mM NaCl treated plants and then declined to the control level in 500 and 750 mM NaCl treated plants (**Figures 2A,B**). There was no significant difference in stem biomass between control and 250 mM NaCl treated plants. However, both fresh and dry biomass of stem decreased significantly in 500 and 750 mM NaCl treated plants as compared to control (**Figures 2C,D**). On the contrary, both fresh and dry biomass of root remained unchanged at all levels of salinity (**Figures 2E,F**). The root/shoot ratio of fresh biomass declined by 23% in 250 mM NaCl treated plants as compared to control, whereas it remain unchanged at high salinity (500–750 mM NaCl) (**Figure 2G**). However, the root/shoot ratio of dry biomass remain unchanged at all levels of salinity (**Figure 2G**). The relative water content (RWC %) of leaf was enhanced significantly at all level of salinity over the control plant by 31.8, 34.9, 35.9 respectively in 250, 500, and 750 mM treated plants (**Figure 2H**).

### Effects of Salinity on Mineral Ion Contents

In the present study, the levels of various mineral ion contents were analyzed to get an insight into the effects of salinity on mineral ion uptake and their accumulation in leaf, stem, and root. Na^+^ content of leaf, stem and root increased progressively with increasing salt concentration (**Table 1**). Na^+^ was found to be accumulated maximum in leaf, but the amount was almost same in stem and root. Although the extent of increase was higher in stem as compared to leaf and root. After 21 days of NaCl treatment, the Na^+^ content of leaf increased by 2, 7.7 and 8.3 folds in 250, 500, and 750 mM NaCl treated plants with respect to control (**Table 1**). In stem, the amount of Na^+^ was elevated by 4.0, 6.5, and 10-folds respectively in 250, 500, and 750 mM NaCl concentration. Likewise in root Na^+^ was increased by 2.8, 4.4, and 14 folds respectively in 250, 500, and 750 mM treated plants. There was no significant changes in K^+^ content of leaf, stem, and root with increasing salinity in *S. persica*. On the other hand, we found maximum Ca^2+^ content in leaf than the stem and root, but there was no significant changes in Ca^2+^ content with increasing salinity in case of stem and root. However, in leaf Ca^2+^ content was decreased significantly in 250 and 750 mM treated plants and found maximum in 500 mM NaCl treated plants as compared to control. Similarly, Mg^2+^ content of leaf, stem, and root was unaffected at all levels of salinity but found maximum in leaf (**Table 1**). The level of various micronutrients (Fe^2+^, Zn^2+^, Mn^2+^, Cu^2+^, and B^2+^) in leaf, stem and root differ markedly in NaCl treated plants as compared to their respective controls. The Fe^2+^ content of leaf and stem remained unchanged at all levels of salinity. However, it declined significantly in root (**Table 1**). Apparently, Fe^2+^ content was markedly high in root than the leaf and stem. Apart from Fe^2+^ content the amount of Zn^2+^, Mn^2+^, Cu^2+^, and B^2+^ were found maximum in leaf samples than the root and stem. There was no significant changes in Zn^2+^ content of leaf and Cu^2+^ content of both leaf and stem with increasing salinity. In stem, the Zn^2+^ content was increased in 750 mM treated plants as compared to control. However, in root the content of Zn^2+^ was decreased with increasing salinity up to 500 mM treatment, but at highest treatment (750 mM NaCl), it was found to be increased as compared to control. Similarly, Cu^2+^ content in the root of *S. persica* was declined in the salt treated plants as compared to control. Maximum amount of B^2+^ was found in leaf as compared to stem and root. However, in root the content was very negligible (**Table 1**).

**Table 1:** Effects of salinity on mineral ion contents of leaf, stem and root of *Salvadora persica* seedlings.

Plant	NaCl	Na^+^	K^+^	Ca^2+^	Mg^2+^	Fe^2+^	Zn^2+^	Mn^2+^	Cu^2+^	B
tissue	(mM)	(mg g^-1^ DW)	(mg g^-1^ DW)	(mg g^-1^ DW)	(mg g^-1^ DW)	(μg g^-1^ DW)	(μg g^-1^ DW)	(μg g^-1^ DW)	(μg g^-1^ DW)	(μg g^-1^ DW)
Leaf	0	8.5 ± 0.8^c^	8.7 ± 2.6^a^	16.1 ± 2.1^a^	2.3 ± 0.5^a^	0.08 ± 0.02^a^	18.5 ± 4.2^a^	41.8 ± 14.6^a^	3.4 ± 0.1^a^	48.4 ± 8.6^a^
	250	17.8 ± 2.2^b^	9.5 ± 2.1^a^	9.6 ± 0.5^b^	1.5 ± 0.3^bc^	0.09 ± 0.02^a^	14.6 ± 2.8^a^	22.7 ± 5.2^b^	3.8 ± 0.06^a^	42.4 ± 8.9^a^
	500	65.8 ± 23.0^a^	10.1 ± 2.1^a^	17.8 ± 1.7^a^	2.0 ± 0.1^ab^	0.1 ± 0.03^a^	18.7 ± 3.6^a^	28.6 ± 2.7^ab^	3.6 ± 1.4^a^	54 ± 15.8^a^
	750	70.5 ± 22.5^a^	8.9 ± 2.5^a^	10.9 ± 4.0^b^	1.3 ± 0.4^c^	0.08 ± 0.03^a^	16.2 ± 4.4^a^	20.8 ± 8.7^b^	3.8 ± 1.0^a^	44 ± 17.6^a^
Stem	0	1.3 ± 0.3^c^	5.3 ± 1.6^b^	2.5 ± 0.1^b^	0.3 ± 0.07^a^	32.4 ± 6.0^a^	8.1 ± 1.4^ab^	6.6 ± 1.1^a^	0.8 ± 0.2^a^	0.5 ± 0.3^b^
	250	5.3 ± 1.6^b^	6.1 ± 1.0^b^	2.7 ± 0.3^b^	0.3 ± 0.02^a^	41.1 ± 18.0^a^	5.6 ± 3.3^b^	3.7 ± 0.9^b^	0.9 ± 0.1^a^	1.0 ± 0.8^b^
	500	8.5 ± 1.2^b^	7.6 ± 1.0^ab^	2.9 ± 0.3^ab^	0.4 ± 0.1^a^	45.6 ± 16.6^a^	6.8 ± 2.8^b^	4.5 ± 1.4^b^	0.9 ± 0.2^a^	0.8 ± 0.4^b^
	750	12.9 ± 4.0^a^	10.3 ± 3.6^a^	3.3 ± 0.7^a^	0.4 ± 0.1^a^	46.7 ± 12.2^a^	12.0 ± 2.8^a^	5.4 ± 1.3^ab^	1.0 ± 0.1^a^	4.4 ± 3.0^a^
Root	0	2.0 ± 0.3^c^	8.6 ± 3.2^a^	0.8 ± 0.3^a^	0.5 ± 0.1^a^	800 ± 193^a^	12.3 ± 5.7^a^	35.2 ± 21.5^a^	3.6 ± 0.7^a^	0.1 ± 0.09
	250	5.6 ± 2.0^bc^	5.8 ± 2.2^a^	1.0 ± 0.4^a^	0.5 ± 0.1^a^	142 ± 54^c^	8.3 ± 1.7^b^	2.2 ± 1.0^b^	1.5 ± 0.4^b^	ND
	500	8.8 ± 1.5^b^	5.9 ± 1.2^a^	1.2 ± 0.6^a^	0.6 ± 0.1^a^	212 ± 50^c^	8.7 ± 1.5^b^	3.4 ± 0.6	1.8 ± 0.4^b^	ND
	750	13.9 ± 7.8^a^	6.6 ± 3.7^a^	1.5 ± 0.9^a^	0.7 ± 0.4^a^	428 ± 174^b^	17.0 ± 4.6^a^	5.5 ± 3.0	2.5 ± 1.2^b^	ND

*The values are mean ± SD (n = 5). Means followed by different letters are significantly different at P ≤ 0.05.*

### Anatomical Modifications Associated with High Salinity

**FIGURE 3:**
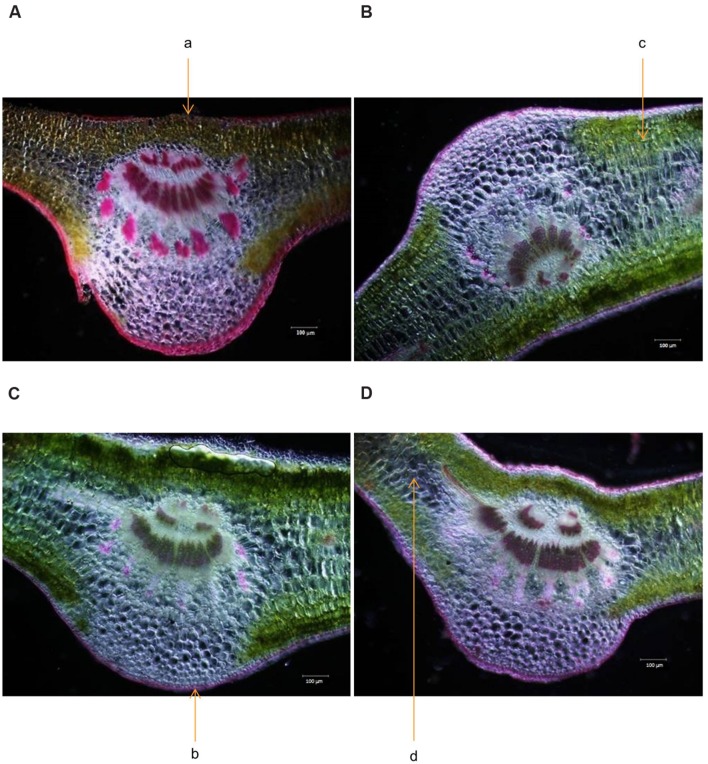
**Effects of salinity on leaf anatomy (10X magnification) of *S. persica* seedlings treated with various levels of salinity. (A)** Control (0 mM); **(B)** 250 mM; **(C)** 500 mM and **(D)** 750 mM NaCl. The different letters in the figure represents: a- upper epidermal cells; b- lower epidermal cells; c- palisade tissue and d- spongy parenchyma tissue.

**FIGURE 4:**
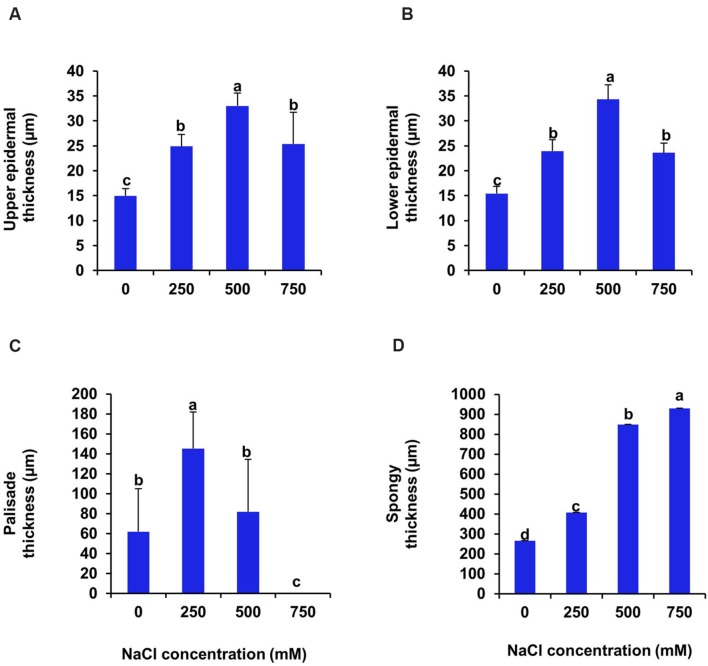
**Effects of salinity on anatomical parameters of leaf. (A)** Upper epidermal; **(B)** Pallisade; **(C)** Spongy parenchyma and **(D)** Lower epidermal thickness of *S. persica* seedlings after 21 days of treatment under hydroponic condition. The values are mean ± SD (*n* = 4). The different letters on the top of the error bars indicate statistically different means at *P* ≤ 0.05.

Cross section of leaf, stem and root of *S. persica* were analyzed to assess the effects of various salt concentration and the anatomical adaptations of this halophyte to be acclimatized under external salinity. There was significant alterations in anatomical features of leaf, stem and root of *S. persica* seedlings imposed to various levels of salinity. By examining the transverse sections of leaf from control and treated samples, it was observed that thickness of upper epidermal layer was increased by 40, 54, and 41% and thickness of lower epidermal layer was increased by 35, 55, and 34% respectively in 250, 500, and 750 mM NaCl-treated plants as compared to control (**Figures 3** and **4A,B**). On the other hand, thickness of palisade tissue layer of leaf of *S. persica* was increased by 57% at low salinity (250 mM NaCl) whereas at moderate salinity (500 mM NaCl) it increased by 24.5% (**Figures 3** and **4C**). Surprisingly, at 750 mM NaCl treatment the palisade layer disappeared in leaf of *S. persica* (**Figure 4C**). In addition, thickness of elongated and densely arranged spongy tissue was also found to rise gradually with increasing salinity. The spongy thickness increased by 35, 69, and 71% respectively in leaf of 250, 500, and 700 mM NaCl treated plants as compared to control (**Figure 4D**).

**FIGURE 5:**
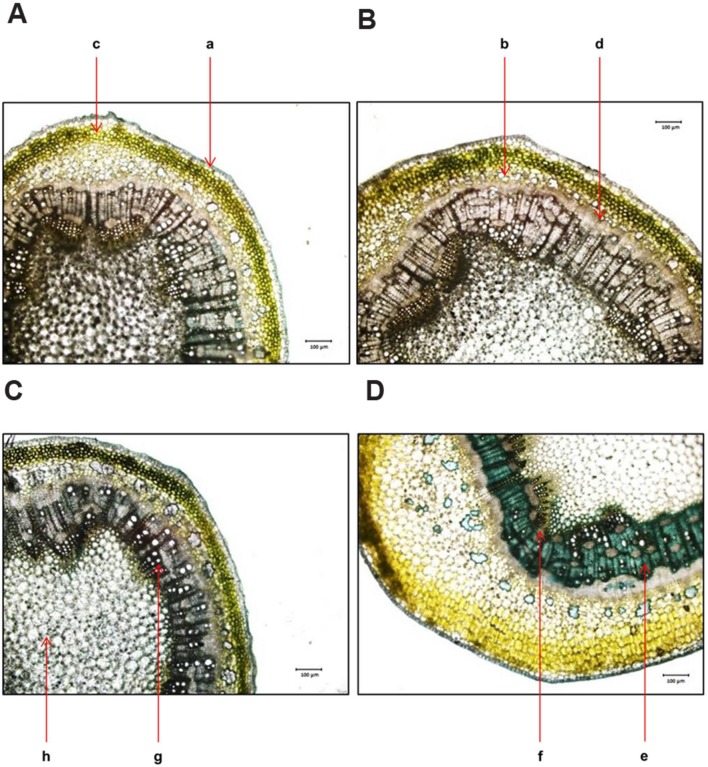
**Effects of salinity on stem anatomy (10X magnification) of *S. persica* seedlings treated with various levels of salinity. (A)** Control (0 mM); **(B)** 250 mM; **(C)** 500 mM and **(D)** 750 mM NaCl. The different letters in the figure represents: a-epidermis; b- hypodermis; c- cortex; d- Endodermis; e- vascular bundle; f- phloem; g- xylem and h- pith.

**FIGURE 6:**
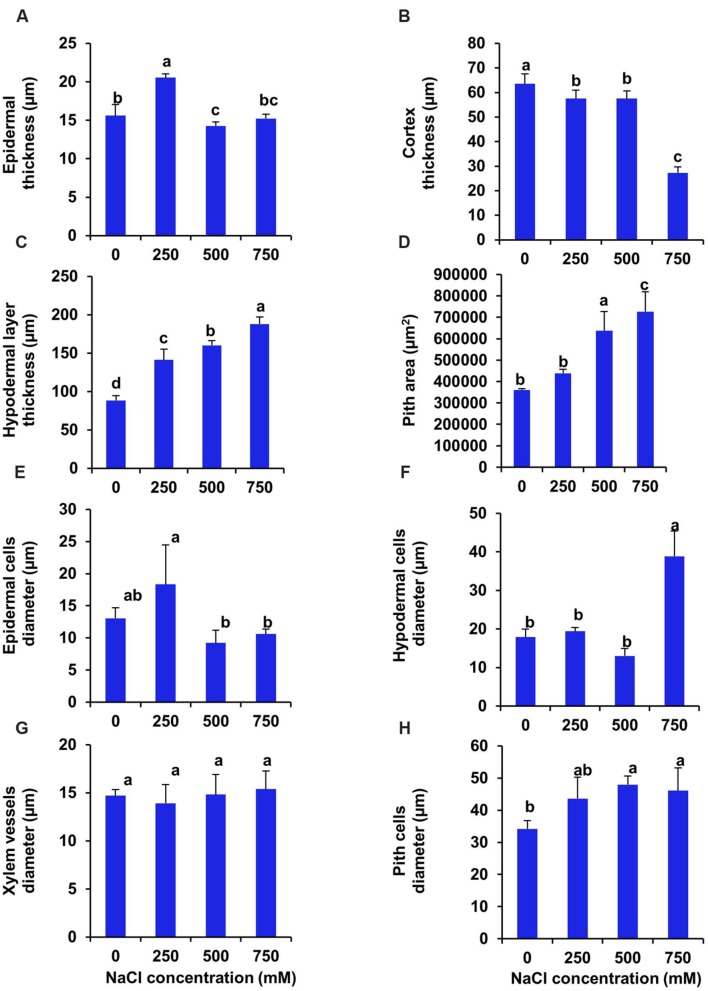
**Salinity induced modifications in stem tissue of *S. persica* seedlings treated with various levels of salinity. (A)** Epidermal layer; **(B)** Cortex layer; **(C)** Hypodermal layer; **(D)** Pith area; **(E)** Epidermal cells diameter; **(F)** Hypodermal cells diameter; **(G)** Xylem vessels diameter and **(H)** Pith cells diameter. The different letters on the top of the error bars indicate statistically different means at *P* ≤ 0.05.

Transverse section of stem of *S. persica* showed 32% increase in thickness of upper epidermal layer at low salinity (250 mM NaCl) whereas, at moderate (500 mM NaCl) and high (750 mM NaCl) salinity there was no significant changes in thickness of upper epidermal layer as compared to control (**Figures 5** and **6A**). The thickness of cortex layers of stem was reduced by 57% in 750 mM NaCl treated plants as compared to control. However, the thickness of cortical layer declined marginally by 9.5% in stem of 250 and 500 mM NaCl treated plants (**Figure 6B**). The thickness of hypodermal layer and pith area of stem were increased progressively with increase in salinity (**Figures 6C,D**). As compared to control, the thickness of hypodermal layer increased by 59, 80, and 112% respectively in stem of 250, 500, and 750 mM NaCl treated plants (**Figure 6C**). Likewise, the pith area increased by 21, 76, and 101% respectively in 250, 500, and 750 mM NaCl treated plants in comparison to control (**Figure 6D**). The epidermal cell diameter of the stem was maximum in 250 mM NaCl treated plants as compared to control and then decreased at higher salinity (**Figure 6E**). The diameter of epidermal cells increased by 40% in stem of 250 mM NaCl treated plants as compared to control and remained unchanged at higher salinity (500–750 mM NaCl). The diameter of hypodermal cells of stem remained unchanged at low (250 mM NaCl) and moderate salinity (500 mM NaCl) as compared to control, but it suddenly increased by 117% at high salinity (750 mM NaCl) treated plants (**Figure 6F**). There was no significant changes observed in the xylem vessels diameter of the stem of *S. persica* (**Figure 6G**). The pith cell diameter increased significantly in stem of salt treated seedlings as compared to control (**Figure 6H**).

**FIGURE 7:**
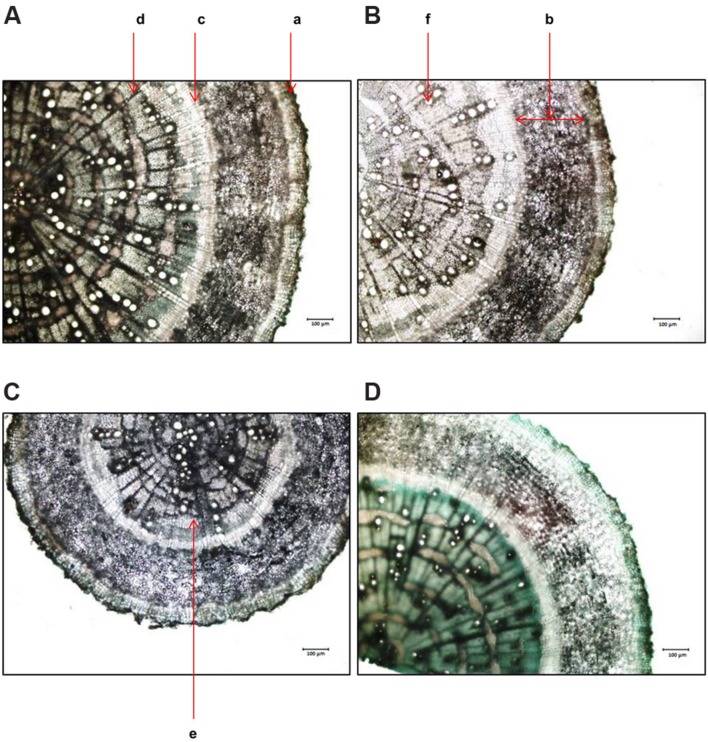
**Effects of salinity on root anatomy (10X magnification) of *S. persica* seedlings treated with various levels of salinity. (A)** Control (0 mM); **(B)** 250 mM; **(C)** 500 mM and **(D)** 750 mM NaCl. The different letters in the figure represents: a- root exhibiting epidermis; b- cortex; c- endodermis; d- vascular bundle; e- phloem and f- xylem.

**FIGURE 8:**
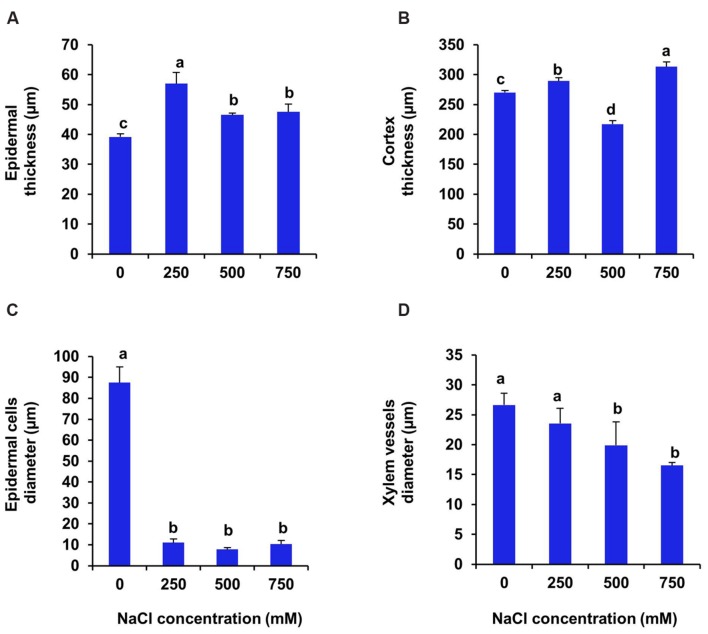
**Salinity induced modifications in root tissue of *S. persica* seedlings treated with various levels of salinity. (A)** Epidermal layer; **(B)** Cortex layer; **(C)** Epidermal cells diameter and **(D)** Xylem vessels diameter. The different letters on the top of the error bars indicate statistically different means at *P* ≤ 0.05.

In case of root the upper epidermal thickness increased by 47, 19.7, and 22.5% respectively, in 250, 500, and 750 mM NaCl treated plants in comparison to control (**Figures 7** and **8A**). However, there was no significant changes in upper epidermal thickness between 500 and 700 mM NaCl treated plants. The cortex thickness was increased marginally at low salinity (250 mM NaCl). However, at moderate salinity (500 mM NaCl), the cortex thickness was reduced by 19% as compared to control (**Figure 8B**). But at high salinity (750 mM NaCl) it was increased by 16% with respect to control. In root of *S. persica*, the epidermal cells diameter was severely affected by salinity. Diameter of epidermal cell of root was decreased by 87.3 % in treated plants and no significant change in root epidermal cell diameter was evident among the treatments (**Figure 8C**) Likewise, the xylem vessels diameter also decreased gradually by 11.6, 25.5, and 31% at 250, 500, and 750 mM NaCl-treated plants as compared to control (**Figure 8D**).

**FIGURE 9:**
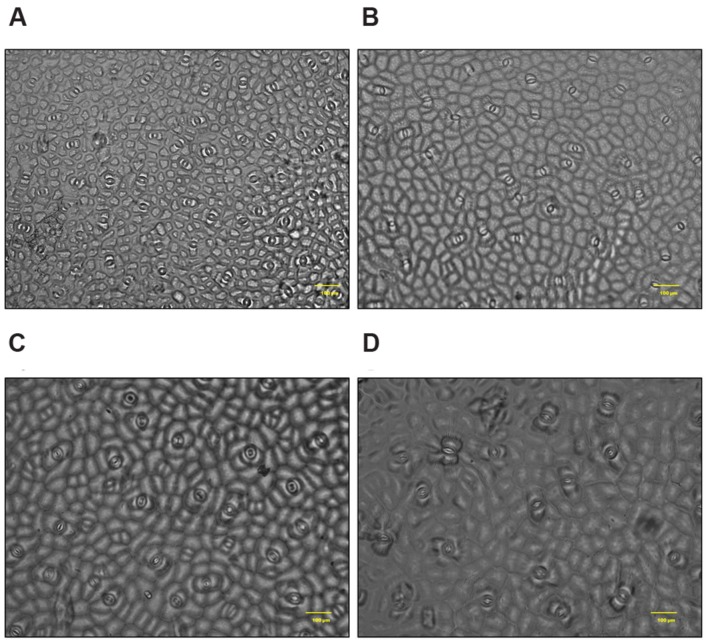
**Salinity induced alterations in stomatal distribution in upper epidermis in leaf of *S. persica* seedlings treated with various levels of salinity (20X magnification). (A)** Control (0 mM); **(B)** 250 mM; **(C)** 500 and **(D)** 750 mM NaCl concentration.

**FIGURE 10:**
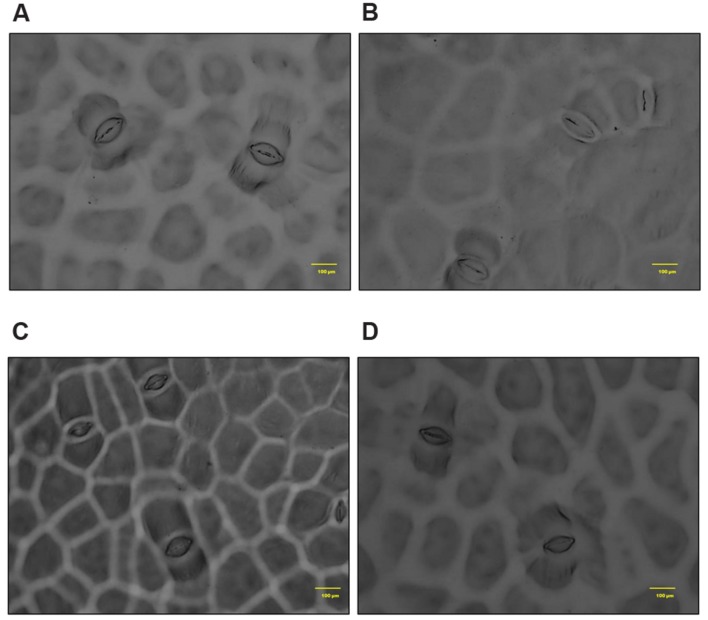
**Salinity induced alterations in stomatal aperture in upper epidermis in leaf of *S. persica* seedlings treated with various levels of salinity (100X magnification). (A)** Control (0 mM); **(B)** 250 mM; **(C)** 500 and **(D)** 750 mM NaCl concentration.

In *S. persica*, the stomatal density on both upper and lower epidermis of leaf decreased sharply in NaCl treated plants as compared to control (**Figures 9** and **10**; **Table 2**). Our study showed that on upper epidermis, stomatal density decreased by 50, 65.8, and 79% respectively in 250, 500, and 750 mM NaCl-treated plants. Similarly, on lower epidermis, stomatal density decreased by 55, 65, and 80.5% respectively, in 250, 500, and 750 mM NaCl treated plants (**Table 2**). In contrary, the stomatal aperture diameter and stomatal pore area showed an inductive response to low level salinity. Both stomatal diameter and aperture area were increased at 250 mM NaCl treatment and then decreased with increasing degree of salinity (**Figure 10**; **Table 2**). However, there was no significant change in SI in leaf of *S. persica* at all levels of salinity (**Table 2**).

**Table 2:** Salinity induced changes in stomata in leaf of *Salvadora persica* seedlings treated with various levels of salinity.

NaCl (mM)	Stomatal density on adaxial surface (Number/μm^2^)	Stomatal density on abaxial surface (Number/μm^2^)	Stomatal Index (%)	Aperture diameter (μm)	Stomatal pore area (μm^2^)
0	82 ± 3.605^a^	67 ± 5.567^a^	5.93 ± 0.24^a^	14.3 ± 1.11^b^	1599.3 ± 89.2^b^
250	41 ± 3.785^b^	30 ± 2.081^b^	5.44 ± 0.47^a^	24.6 ± 2.59^a^	2361.7 ± 328.0^a^
500	28 ± 3.511^c^	23 ± 2.516^c^	6.55 ± 0.75^a^	6.40 ± 1.10^c^	476.0 ± 56.2^c^
750	17 ± 2.516^d^	13 ± 1.527^d^	5.06 ± 0.69^a^	3.70 ± 1.10^d^	368.4 ± 65.3^cd^

*The values are mean ± SD (n = 10). Means followed by different letters are significantly different at P ≤ 0.05.*

### Effects of Salinity on Total Sugar, Reducing Sugar and Starch Contents

**FIGURE 11:**
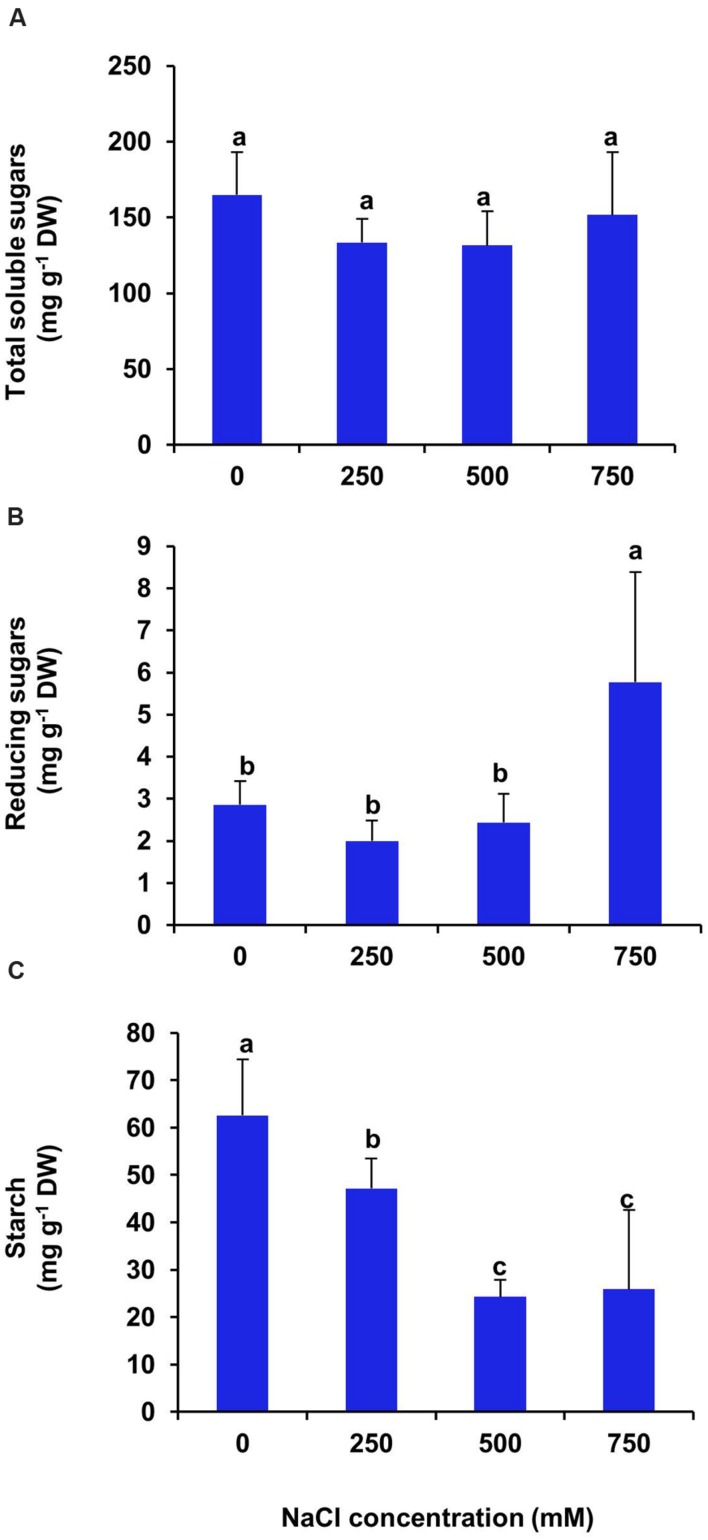
**Salinity induced changes in carbohydrate contents of *S. persica* leaf. (A)** Total soluble sugars; **(B)** Reducing sugars and **(C)** Starch. The values are mean ± SD (*n* = 5). The different letters on the top of the error bars indicate statistically different means at *P* ≤ 0.05.

In *S. persica*, the carbohydrate contents *viz*., total sugar, reducing sugar and starch, were diversely affected by the NaCl treatment. There was no significant changes in total soluble sugar content at all levels of salinity (**Figure 11A**). On the contrary, the reducing sugar content remained unchanged at low and moderate salinity (250–500 mM NaCl) as compared to control (**Figure 11B**). However, it increased by twofolds in 750 mM NaCl treated plants of *S. persica* (**Figure 11B**). After 21 days of treatment, the starch content of leaf decreased gradually with increasing salinity. However, there was no significant difference in starch content between moderate (500 mM) and high (750 mM) salt treated plants (**Figure 11C**). After 21 days of treatment, the starch content of leaf decreased by 33, 61, and 59% respectively in 250, 500, and 750 mM NaCl treated plants as compared to control (**Figure 11C**).

### Effects of Salinity on Total Free Amino Acids, Proline and Polyphenol Content

**FIGURE 12:**
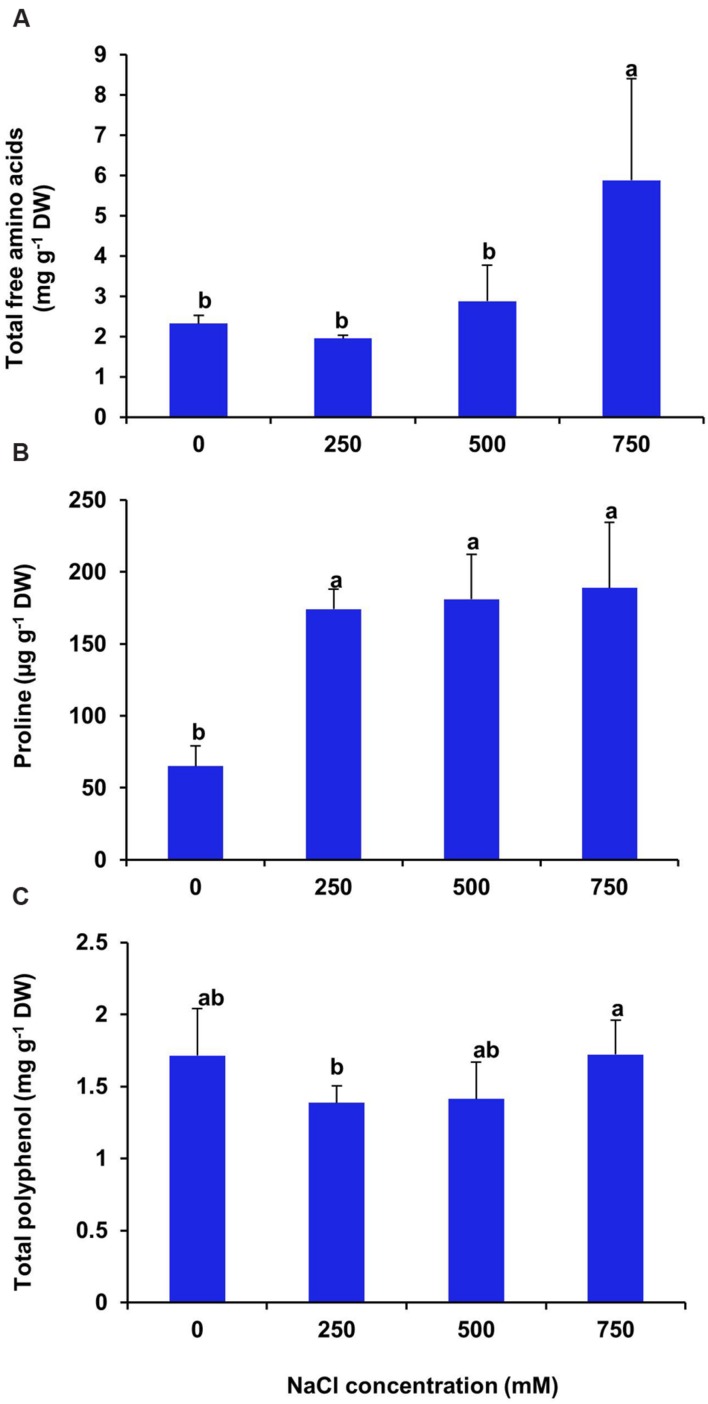
**Effects of salinity on some organic metabolites. (A)** Total free amino acids; **(B)** Proline and **(C)** Total polyphenol contents in leaf of *S. persica* seedlings after 21 days of salt treatment. The values are mean ± SD (*n* = 5). The different letters on the top of the error bars indicate statistically different means at *P* ≤ 0.05.

There was no significant changes in total free amino acid content of leaf at low and moderate salinity as compared with control (**Figure 12A**), whereas, at high salinity (750 mM NaCl) treatment the free amino acid content increased significantly by 2.5 folds in respect to control. In the present investigation, we found an increase in proline level by 2.6, 2.7, and 2.9 folds respectively in the plants imposed to 250, 500, and 750 mM NaCl treatments as compared to control (**Figure 12B**). In *S. persica*, there was no significant changes in total polyphenol content of leaf at all levels of salinity (**Figure 12C**).

## Discussion

Salinity profoundly affects growth of both glycophytes and halophytes. The degree of salt tolerance varies extensively between different plant species. Halophytes commonly possess more tolerance to salt environment than the glycophytes (Yu et al., 2011; Kosová et al., 2013). In the present investigation, the seedlings of *S. persica* showed optimum growth at 250 mM NaCl concentration. Higher salinities (500–750 mM) caused significant reduction in growth of *S. persica*, but *S. persica* plant was able to grow and survive up to 750 mM NaCl without any visible symptoms of leaf wilting. The decline in growth at supra-optimal salinity (500–750 mM NaCl) was associated with a marked decrease in leaf area. The reduction in growth can save energy cost, reduce ROS production, decrease amino acid demand for protein synthesis, and thereby provide more free amino acids for osmotic adjustment (Yu et al., 2011). In spite of higher accumulation of sodium in leaf tissue with increasing salinity, the RWC % of leaf did not decline rather it increased by high salinity and there was no visible symptoms of leaf injury. The above results provide an indirect but strong indication that Na^+^ is effectively compartmentalized in the vacuoles and this is required for osmotic adjustment. This hypothesis is further reinforced by the fact that both leaf succulence (water content per unit area) and leaf thickness (data not shown) were considerably higher in the leaves of high salt treated plants.

Water relations and the ability to adjust the osmotic concentration play a vital role in overall growth and development of plants (Munns, 2003). In the present investigation, salinity treatment caused a progressive increment in leaf water content, thereby increased the leaf turgor. The capacity of *S. persica*, to maintain high RWC in their leaves, despite of high external salinity might have a protective role from the deleterious effects of salinity. Tissue tolerance of salinity requires compartmentalization of toxic sodium ion at the cellular and intracellular levels to avoid toxic concentrations in the cytoplasm, especially in mesophyll cells of leaves (Munns and Tester, 2008; Wang et al., 2015). Increased leaf succulency is a typical adaptive response in halophytes and may be achieved by increasing the size of mesophyll cells and the relative size of their vacuoles (Shabala and Mackay, 2011). The increased leaf succulence in *S. persica* might be to counter the increased Na^+^ ion content from the cytosol of the leaf tissue. The key driving force between the succulency phenomenons is plant’s attempt to increase the size of its vacuole, to sequester large quantities of Na^+^ away from metabolic active compartments of the cell (Shabala, 2013).

Adaptation of plants under high salinity involves changes in uptake and transport of mineral ions to maintain cellular homeostasis. The maintenance of shoot osmotic and turgor pressure under saline conditions is predominantly achieved in halophytes by using inorganic ions (Na^+^, Cl^-^ and K^+^) to maintain shoot osmotic and turgor pressure under saline conditions, while glycophytes accomplish this largely by increased *de novo* synthesis of compatible solutes (Shabala, 2013; Shabala and Pottosin, 2014). The three major inorganic ions such as Na^+^, K^+^ and Cl^-^ account for 80–95% of the cell sap osmotic pressure in halophytes (Shabala and Mackay, 2011; Shabala, 2013). In halophytes, the maintenance of cell turgor pressure under hyperosmotic saline conditions is achieved by the efficient sequestration of these cytotoxic ions in vacuoles. Such sequestration, however, requires a synchronized increase in the osmotic potential of the cytosol, and cytosolic K^+^ retention appears to be absolutely essential for this process (Hariadi et al., 2011; Shabala and Pottosin, 2014). As evidenced from our study, accumulation of Na^+^ ion increased parallel with the increase in NaCl concentrations in leaf, stem and root of *S. persica*, while the level of K^+^, Ca^2+^, Mg^2+^ did not change significantly with increasing degree of salinity. The halophytic plants are able to take advantage of the close similarity between Na^+^ and K^+^, and have adapted to grow in areas of high salinity. The presence of Na^+^ in the environment and its uptake by plants can reduce the amount of K^+^ required to meet the plant’s basic metabolic process (Benito et al., 2014). In *S. persica*, Na^+^ content of leaf was found to be much higher than the stem and root. This strongly suggests that Na^+^ is translocated to the leaf tissue for osmotic adjustment purposes. The preservation of cell turgor pressure is very sensitive to a limited K^+^ supply and because of its high mobility, K^+^ is usually the principal cation that contributes to vacuole and cell expansion (Benito et al., 2014). It has been proposed that as total tissue K^+^ concentration drops, the cytoplasm maintains a homeostatic concentration of K^+^ to enable K-dependent processes (Benito et al., 2014). It has been reported that intracellular potassium homeostasis is a prerequisite for the optimal operation of plant metabolic machinery and plant’s overall performance (Shabala and Pottosin, 2014). Our results also suggest that homeostatic concentration of K^+^ is maintained in the cytosol of *S. persica* plants imposed with high salinity. It has been suggested that the effects of divalent cations (Ca^2+^ and Mg^2+^) on Na^+^ eﬄux is transient, while they cause a prolonged shift toward K^+^ uptake. It has also been proposed that in addition to their known ability to block non-selective cation channels (NSCC) responsible for Na^+^ entry, divalent cations also regulate the activity of K^+^ transporters at the plasma membrane of mesophyll cells, thereby maintaining the high K^+^/Na^+^ ratio required for optimal leaf photosynthesis (Shabala et al., 2005; Pérez et al., 2008). In *S. persica*, higher leaf Na^+^ content was also harmonized with higher levels of Ca^2+^ and Mg^2+^ contents of leaf than in roots, while K^+^ content was almost similar in leaf and root. This result may be indicative of the important role of divalent cations (Ca^2+^ and Mg^2+^) as blockers of outward-rectifying K^+^ eﬄux channels, enabling efficient K^+^ retention in photosynthetically active shoot tissues (Shabala et al., 2005; Pottosin and Shabala, 2015).

Anatomical modification of leaf, stem, and root was observed in *S. persica* seedlings treated with various levels of salinity. The thickness of both upper and lower epidermis was increased in leaf of salt treated seedlings as compared to control. This increase in epidermal thickness in salinity might be an adaptation of this halophyte to minimize the transpiration rate and hence to maintain the water content in mesophyll tissue. Salinity induced increase in epidermal thickness not only improve the water use efficiency (WUE) of plants but also provide additional space for efficient sequestration of Na^+^ in the leaf epidermis (Shabala et al., 2012). At higher saline condition (750 mM NaCl) the epidermal thickness declined to the value detected in leaf of 250 mM NaCl treatment and this decrease in epidermal thickness may be attributed to the limited cell division and growth at higher salinity (Carcamo et al., 2012). In contrast to our study, the epidermal thickness was declined in the glycophyte *Hordeum vulgare* with increasing salinity (Atabayeva et al., 2013) which suggests that there is differential anatomical adaptation between halophyte and glycophyte in response to salinity. The palisade tissues are the chlorenchymatous mesophyll tissue which contains numerous chloroplasts and considered principal site for photosynthesis. The palisade tissue thickness in the leaf of *S. persica* increased at low salinity (250 mM NaCl), then decreased to the control level at higher salinity (500 mM NaCl) and interestingly disappeared in leaf of 750 mM NaCl treated plants. In contrasts to our results there was a gradual increase in thickness of palisade tissue with increasing salinity in semi-mangrove plant *Myoporum bontioides* (Xu et al., 2014). The significant decrease in the palisade tissue at extreme salinity (750 mM NaCl) might be an adaptation of this halophyte to minimize the photosynthetic energy utilization in the higher saline condition. In *S. persica*, the spongy tissue thickness increased with increasing salinity. Enhanced spongy tissue in salt treated seedlings of *S. persica* helps in maintaining leaf water content and turgor. Increase in spongy tissue of *S. persica* might be contributed to increased leaf succulence under high salinity condition. Distribution of stomata on the leaves of *S. persica* along with stomatal aperture diameter and pore size was also affected by salinity. The stomatal density and pore size decreased on both adaxial and abaxial surfaces in leaf of *S. persica*. Decrease in stomatal density with increasing salinity have also been reported in several other halophytes such as *Bruguiera parviflora* (Parida et al., 2004a); *Nitraria retusa and Atriplex halimus* (Boughalleb et al., 2009) and *Chenopodium quinoa* (Shabala et al., 2012, 2013). In *S. persica*, salinity did not cause significant changes in SI. This result suggests that decrease in stomatal density in *S. persica* may be due to increased leaf succulency and an increase in the size of pavement cells. It could be suggested that by doing so *S. persica* plants not only improve its WUE but also provide additional space for Na^+^ sequestration in the leaf epidermis (Shabala et al., 2012). It has been suggested that the observed reduction in stomatal density might represent a fundamental mechanism by which a plant may optimize WUE under saline condition (Adolf et al., 2013; Shabala et al., 2013).

Salinity induced significant alternation in stem anatomy of *S. persica* seedlings treated with various levels of salinity. The hypodermal thickness and pith area increased with increase in salinity which may be a defensive attribute under salinity condition. The thickness of cortex decreased in stem of *S. persica* with increase in salinity. The decrease in the stem cortical cell thickness is mainly due to the collapse of the cortical cells in response to severe salinity (Alam et al., 2015). This may be beneficial to curtail growth under salinity conditions by conserving essential energy for survival (Alam et al., 2015; Nawaz et al., 2016). There was no significant changes in xylem vessel diameter in stem of *S. persica* with increasing salinity. This may be considered as an adaptive mechanism of *S. persica* to maintain a steady water flow in shoot even at high saline condition and with steady water flow may help in translocation of more mineral ions to the shoot. In contrasts to our results, salinity induced decrease in xylem vessels diameter causing decrease in water and mineral conductivity have been reported in many plants (Sandalio et al., 2001; Ortega et al., 2006; Rewald et al., 2012; Atabayeva et al., 2013). Plant roots are the first line of damage or first line of defense due to the direct encounter with the saline soil solution (Rewald et al., 2012). Unlike shoot, the diameter of root vascular bundle decreased with the increase in salinity treatment. It has been reported that xylems of plants encountering salinity are tends to have vessels with lower diameter than their unstressed counterpart (Junghans et al., 2006). Decrease in the vessel diameter can be attributed to reduce the hydraulic conductivity in the part formed during the stress period (Junghans et al., 2006). This modification in root xylem of the stressed plant confers safety to the vessel with considerable conductivity (Junghans et al., 2006). However, by reducing the vessel diameter of root, water uptake also reduced with increasing salinity and hence reduces photosynthesis and growth of the plant. Increase in the root cortical cell thickness under saline condition was also reported in the xero-halophyte shrubs *Nitraria retusa* and *Atriplex halimus* (Boughalleb et al., 2009). Salinity induced increase in epidermal thickness of root reduces salt ion diffusion in to the root.

The primary physiological response of plants under high saline environment is to undergo osmotic adjustment through two processes: ions accumulation in the vacuole and synthesis of compatible solutes in the cytosol (Li et al., 2010). Therefore, salinity induced changes in levels of various organic metabolites such as total sugar, reducing sugar, starch, total free amino acids and proline were analyzed to decipher the role of these organic metabolites for osmotic adjustment and salt tolerance of *S. persica.* We observed no significant changes in total soluble sugar content in leaves of *S. persica* at all levels of salinity. On the contrary, the reducing sugar content increased significantly at extreme salinity and starch content decrease progressively with increase in salinity. The decreased starch content and the increased reducing sugar content under high saline condition might be due to the starch-sugar inter-conversion to provide more sugar for osmo-protection under high salinity (Parida and Jha, 2013; Slama et al., 2015). In *S. persica*, total free amino acid content increased at extreme salinity (750 mM NaCl) and proline content increased at all levels of salinity as compared to control. The important amino acids include alanine, arginine, glycine, serine, leucine, and valine, together with the proline, and the non-protein amino acids; citruline and ornithine. The increased accumulation of free amino acid might be due to the increased biosynthesis of amino acids or increased activity of protease for osmotic adjustment (Parida et al., 2007). Our result agrees with the previous reports showing accumulation of free amino acids on exposure to salinity stress (Hajlaoui et al., 2010). Proline is an important osmolyte produced at a high cost of energy as compared to uptake of Na^+^ into cytoplasm. The present study showed increased levels of proline content at all levels of salinity. The salinity-induced increase of proline in *S. persica* might be due to promotion of proline biosynthesis and/or may be due to inhibition of proline catabolism (Parida et al., 2008; Huang et al., 2013). Increased level of proline in response to salt stress has been reported in many plants (Moghaieb et al., 2004; Parida et al., 2004b; Koyro, 2006; Rajaravindran and Natarajan, 2012; Zakery-Asl et al., 2014). Proline have some osmo-regulatory function besides it also have some other functions such as membrane protection and stabilization of enzymes (Bandurska, 2000; Hassine et al., 2008; Parida and Jha, 2013). However, in *S. persica*, the reported proline concentration is only up to 200 ug g^-1^ FW, or less than 2 mM on a water basis. In osmotic terms, this is almost nothing compared to high 250–750 mM NaCl concentration in the apoplast. Therefore, osmoregulatory function of proline in *S. persica* is ruled out and increase in total amino acid pool suggest that amino acids other than proline may have role in maintaining osmotic balance of the cell under high salinity condition in *S. persica*. Polyphenols play an important role as non-enzymatic ROS scavengers in plants (Matysik et al., 2002). They protect the plants from oxidative damage by increasing stability of the cell membrane (Burguieres et al., 2007). Our data showed that the polyphenol content remained unaffected by salinity in *S. persica* plant. In contrast to our results, salinity induced higher accumulation of polyphenols has been reported in many plants (Kirakosyan et al., 2004; Ksouri et al., 2007; Yuan et al., 2010). The unchanged level of polyphenols by salinity indicates that polyphenols in *S. persica* are in threshold level to counter the salinity induced oxidative damage.

## Conclusion

In summary, our results suggest that *S. persica* can tolerate extreme salinity by maintaining osmotic balance and ion homeostasis. Salinity induced higher accumulation of organic metabolites such as amino acids, reducing sugars and polyphenols suggests their role in osmotic regulation, ROS scavenging and protection of cellular macromolecules in *S. persica* under high salinity condition. The role of proline in osmoprotection in *S. persica* was ruled out. The decreased biomass suggests that plant incurred high energy cost for synthesis and transportation of organic solutes to maintain osmotic balance and water status under high salinity condition in *S. persica*. Salinity induced reduction in growth of *S. persica* may help in better adaptation under high salinity condition by saving photosynthetic energy, reducing protein synthesis and making more free amino acids available for osmotic adjustment. The amount of Na^+^ increase significantly with increase in salinity whereas K^+^, Ca^2+^, and Mg^2+^ content remained unchanged and the divalent cations (Ca^2+^ and Mg^2+^) were found at higher level in leaf than root. The results suggest the important role of divalent cations (Ca^2+^ and Mg^2+^) as blockers of outward-rectifying K^+^ eﬄux channels, enabling efficient K^+^ retention in photosynthetically active shoot tissues. Salinity induced higher accumulation of Na^+^ content in leaf of *S. persica* suggest that Na^+^ is translocated to the leaf tissue for osmotic adjustment. The increased leaf succulence in *S. persica* might be the plant’s attempt to increase the size of its vacuole, to sequester large quantities of Na^+^ away from metabolic active compartments of the cell. Stomatal density decreased by salinity whereas SI remained unchanged and by this the plant not only improve its WUE but also provide additional space for Na^+^ sequestration in the leaf epidermis. Salinity induced unchanged level of xylem vessel diameter of stem may be considered as an adaptive mechanism of *S. persica* to maintain a steady water flow in shoot under saline condition. From our study, it was concluded that *S. persica* survive under extreme salinity conditions without any lethal effects on plants. Based on the present study the key mechanisms of salt tolerance in this species may be higher accumulation of organic metabolites, increase in leaf succulency, efficient Na^+^ sequestration in the vacuole, K^+^ retention in the photosynthetic tissue and increase in WUE by reducing stomatal density.

## Author Contributions

AK performed most of the experiments and prepared the manuscript. AKP designed and coordinated the experiments, analyzed the data, interpreted the results and improved the manuscript. SV maintained the plants, prepared the media and reagents, gave periodical stress treatments and performed some experiments. PA provide some inputs to improve the manuscript.

## Conflict of Interest Statement

The authors declare that the research was conducted in the absence of any commercial or financial relationships that could be construed as a potential conflict of interest.
